# Quality Control and Regulatory Scientific Research on Collagen‐Based Medical Devices

**DOI:** 10.1111/cpr.70142

**Published:** 2025-11-05

**Authors:** Rui Wang, Jianfeng Shi, Jie Zhou, Linnan Ke, Chongxia Yue

**Affiliations:** ^1^ Division of Biomaterials National Institutes for Food and Drug Control Beijing China; ^2^ China National Accreditation Service for Conformity Assessment Beijing China; ^3^ College of Biomedical Engineering, Institute of Regulatory Science for Medical Devices Sichuan University Chengdu Sichuan China

**Keywords:** collagen, medical devices, quality control

Collagen is a key structural component of the extracellular matrix and is widely present in all tissues and organs, mainly in skin, bones, tendons, ligaments, cartilage, mucous membranes, and so on [1]. About 29 types of collagen have been identified so far. According to their structural characteristics, synthesis methods, biological functions, and so on, they can be roughly divided into seven categories. Figure 1 illustrates the presence of collagen in the human body. Type I, type II, and type III collagen are the most abundant and most widely studied collagens. Among them, type I collagen is the most abundant fibrous collagen in animals, accounting for about 90% of the total collagen in animals. It is widely distributed in the skin, bones, tendons, ligaments and connective tissue interstitium [2]. Type I collagen mainly serves as tissue support to impart tissue tension and maintain the integrity and mechanical properties of bone structures. Besides, it also plays a role in the physiological and pathological processes of cells and tissues [3]. Type II collagen is a fibrous collagen that is very similar in structure to type I collagen. It is primarily composed of three α1(II) chains forming a homotrimer, which is covalently cross‐linked with type XI collagen and associated with glycoproteins to maintain tissue integrity and withstand elastic stress. Type II collagen is the main component of articular cartilage, accounting for 95% of all collagen in cartilage [4]. Therefore, the degradation and formation of type II collagen are vital in the evaluation of cartilage renewal and cartilage repair. Type III collagen is a fibrous collagen composed of three α1(III) chains, that are abundantly expressed in various connective tissues, such as the skin, lungs, intestines, and vascular system. It frequently exhibits co‐localization with type I collagen within these tissues. Type III collagen is essential for maintaining the structural integrity of the skin and the synthesis of type I collagen in the skin [5].

**FIGURE 1:**
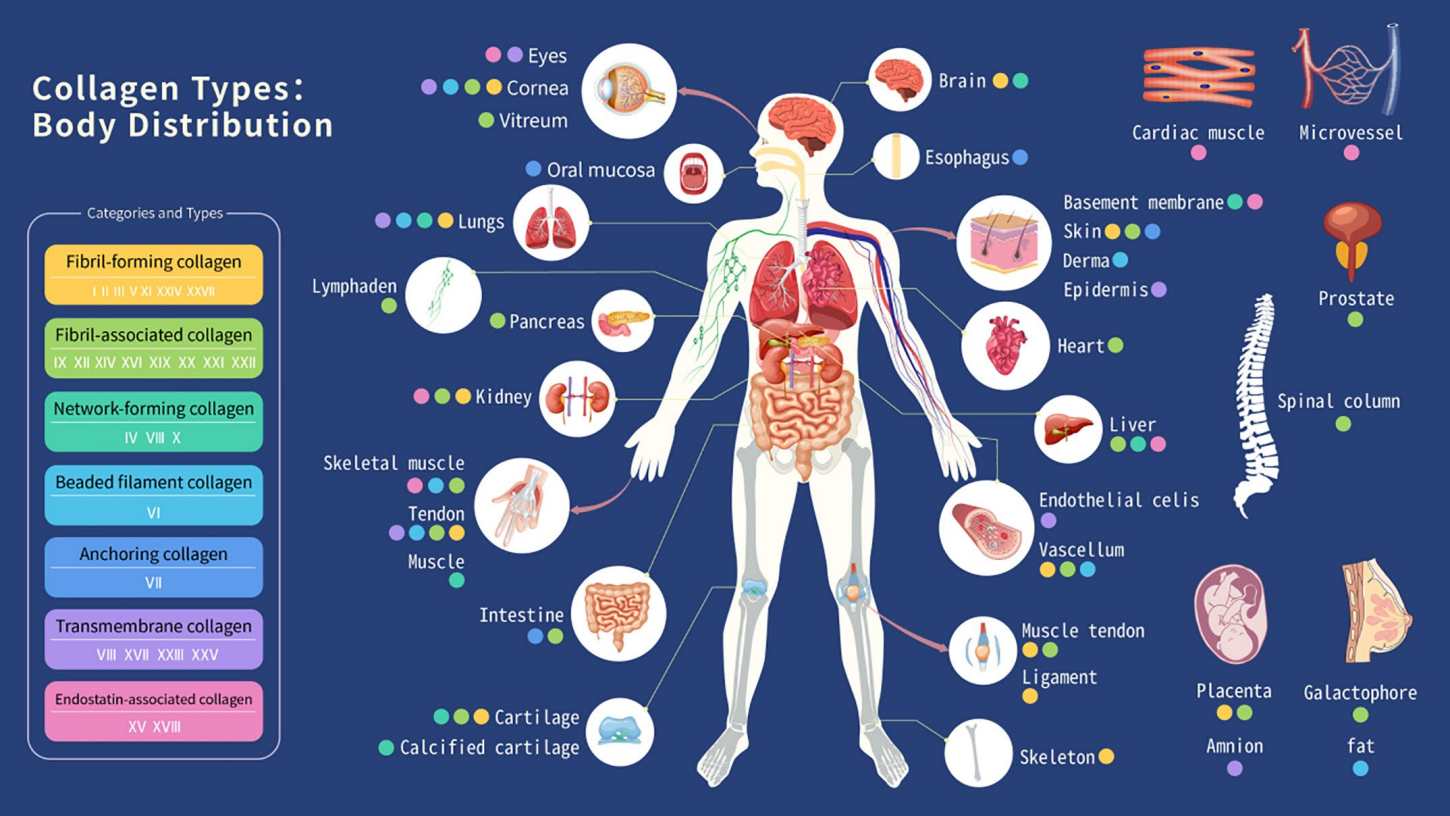
Collagen types and their body distribution.

As a highly abundant and ubiquitously distributed functional protein in the human body, collagen exhibits outstanding properties that make it widely applicable across diverse fields of medical devices. For example, collagen sponges perform well in surgical haemostasis; collagen bone repair materials could provide space for cell adhesion, proliferation and migration, promoting the differentiation and proliferation of bone cells and accelerating bone tissue repair [6]; collagen implants demonstrate significant efficacy in tissue regeneration and filling and gradually become an emerging force in beauty products. Collagen‐based medical devices are available in a variety of forms, including collagen sponges, absorbable sutures, oral guidance films, fillers (skin, cartilage), dressings/patches, decellularized matrix products, and so on. Based on the intended use and related risks, collagen‐based medical devices are typically classified as Class II or III by the National Medical Products Administration (NMPA). Verification of safety and efficacy tailored to specific indications constitutes a fundamental regulatory requirement for collagen‐based medical devices.

Depending on the preparation method, collagen is categorized as animal‐derived, recombinant, or cell‐synthesized (ECM‐like), with products from each source exhibiting significantly different quality control priorities. Animal‐derived collagen is currently widely used in the field of medical devices with various product forms. How to control its quality and safety is of great significance to this type of product. The key points for quality control of animal‐derived collagen final products mainly involve animal‐derived raw materials, preparation processes and intended uses of the products [7]. First of all, animal‐derived raw materials need to meet the requirements of the series standards of GB/T 44353 “Medical devices utilizing animal tissues and their derivatives” and the relevant requirements of the “Technical Review Guidelines for Registration of Animal‐Derived Medical Devices (2017 Revision)” [8]. Medical device registrants need to pay particular attention to the sources of animal‐derived raw materials, the collection and processing of animal tissues and virus inactivation/removal [9]. Researchers conduct comprehensive risk analysis and risk control to ensure that the biological safety and immunogenicity of the final products meet the regulatory requirements. Secondly, control and verify the physical and chemical properties as well as biological properties of collagen. The physical and chemical properties include appearance, physical and chemical properties, crosslinking, pH value, osmotic pressure, molar concentration, collagen identification, content, purity, molecular weight, heat stability, primary structure, advanced structure, impurities (impurity proteins, carbohydrates, endotoxins, microbial limits, heavy metals, trace elements, etc.), viscoelasticity, mechanical properties, and so on. Biological properties include collagen‐cell interaction, degradation properties and biocompatibility evaluation according to the series standards of GB/T 16886 “Biological evaluation of medical devices” [10]. Finally, the stability of the final product, sterilization method and effect should be evaluated. For detailed information, please refer to YY/T 1453–2016, YY/T 0954–2015, YY/T 1794–2021. Recombinant collagen refers to the use of recombinant DNA technology to genetically manipulate and/or modify the gene encoding the required human collagen and use plasmids or viral vectors to introduce the target gene into appropriate host cells (bacteria, yeast or other eukaryotic cells), then expressed and translated into collagen or collagen‐like polypeptides and prepared through steps such as extraction and purification [11]. Currently, there are two most commonly used expression systems in industrialization: the *Bacillus* expression system and the *Pichia pastoris* expression system [12]. Recombinant collagen is generally fermented, extracted and purified to form original liquid, which is then processed into corresponding medical device products according to the intended use. Therefore, based on the preparation principle and preparation process of recombinant collagen, the quality control elements of recombinant collagen products can be summarized as follows: (1) Physical and chemical properties: including appearance, solubility, ignition residue, pH value, moisture content, osmotic pressure, molarity, kinetic viscosity, thermal stability, and so on; (2) Structural characterization: primary structure, advanced structure; (3) Recombinant collagen characteristics: identification, purity, content; (4) Impurities, contaminants and additives: exogenous DNA residues, host protein residues, impurities derived from the culture medium (including inducers (polynucleotides, viruses), antibiotics, serum and other culture medium components), pro‐inflammatory contaminants (peptidoglycan, etc.), heavy metal and trace element content, additives (preservatives, freeze‐drying protectants, processing aids such as enzymes, chemical/biochemical treatment reagents (e.g., cyanogen bromide, guanidine, oxidants and reducing agents), solvents, carriers/ligands (such as monoclonal antibodies) and other filterable substances, etc.); (5) Safety test: sterility, bacterial endotoxin, microbial limit; (6) Stability, biological evaluation, packaging, transportation and storage. For detailed information, please refer to YY/T 1849‐2022 “Recombinant collagen protein”, YY/T 1888‐2023 “Recombinant Humanized Collagen”, and so on. In addition, collagen synthesis via cellular biotechnology represents an emerging production approach. It mainly refers to culturing human cells (including human mesenchymal stem cells, fibroblasts, etc.) on a two‐dimensional or three‐dimensional scaffold and regulating extracellular matrix components by adjusting the composition of the culture medium. After culturing for a certain period, the cells and cell culture scaffolds are removed through decellularization technology and then type I and type III collagen can be purified [13]. The collagen isolated and purified from the extracellular matrix has similar molecular structure and biological properties to animal‐derived collagen. However, the relationship between its stability, purity, molecular structure and biological properties between culture batches needs to be further studied [14]. Therefore, the quality control priority for these products should focus on the controllability of the cell culture passage, the addition of animal‐free additives, the residue of processing aids in the purification process and the content and purity of collagen.

Nowadays, the scale of China's collagen market has shown rapid growth. China's collagen product market was valued at RMB 28.8 billion in 2021, with a staggering year‐on‐year increase of 40.5%, and expanded to approximately RMB 71.9 billion by 2024. There is no doubt that collagen is in a golden period of rapid development and the market growth trend is clear. The strong market demand brings both opportunities and challenges to the research and development of collagen products. Consumers have increasingly higher requirements for the safety and effectiveness of collagen products and manufacturers need to strengthen product development and quality control to ensure the safety and effectiveness of the products. While manufacturers vigorously develop new products and technologies, they must also conduct quality control at every step from raw material production to product launch, in order to minimise potential risks and ensure product safety and effectiveness. Therefore, accurate, scientific and controllable testing is crucial for such products. To better ensure the reliability of test results, the China National Accreditation Service for Conformity Assessment (CNAS) plays a critical role in quality control, regulatory science, international certification and research and development support for collagen‐based medical devices. Through laboratories and testing institutions accredited by CNAS, physicochemical properties of collagen, such as appearance, molecular weight, purity, thermal stability, pH, and osmotic pressure, could be tested to ensure compliance with relevant industry standards. Additionally, accredited laboratories could conduct biocompatibility evaluations, including cytotoxicity, sensitization, irritation and implantation reactions, ensuring that products meet the GB/T 16886 series of standards. For animal‐derived collagen products, CNAS‐accredited laboratories also conduct biosafety tests, such as virus inactivation/removal validation and immunogenicity testing to ensure compliance with the GB/T 44353 series of standards. CNAS not only ensures that the quality of collagen‐based products meets both international and domestic standards, enhancing their market competitiveness, but also guarantees the authenticity and accuracy of testing through supervision and review, which dramatically ensures product quality and safety [15]. As the collagen market flourishes, CNAS will help related enterprises improve product quality and reduce trade barriers through mutual recognition with international accreditation organisations. In general, manufacturers should also strictly control product quality from multiple aspects, including raw material production, process control, finished product quality and so on. With the ongoing advancement of collagen technology, the development of more efficient collagen‐expressing cell factories through genetic engineering or the personalised customization of collagen scaffolds using 3D printing technology will become future R&D trends [16, 17]. Alongside rapid technological progress, establishing a rigorous quality control system and enhancing product competitiveness through standardisation and certification of collagen products will contribute to the healthy and sustainable development of the industry.

## Author Contributions

Rui Wang drafted the manuscript and conducted the literature review. Jianfeng Shi and Jie Zhou provided key points for quality control in the experiments. Linnan Ke and Chongxia Yue reviewed and edited the manuscript, with overall responsibility for its quality assurance. All authors read and approved the final article.

## Conflicts of Interest

The authors declare no conflicts of interest.

## Data Availability

Data sharing not applicable to this article as no datasets were generated or analysed during the current study.
